# Mammogram Image Enhancement Techniques for Online Breast Cancer Detection and Diagnosis

**DOI:** 10.3390/s22228818

**Published:** 2022-11-15

**Authors:** Daniel S. da Silva, Caio S. Nascimento, Senthil K. Jagatheesaperumal, Victor Hugo C. de Albuquerque

**Affiliations:** 1 Department of Teleinformatics Engineering, Federal University of Ceará, Fortaleza 60455-970, CE, Brazil; 2 Department of Electronics and Communication Engineering, Mepco Schlenk Engineering College, Sivakasi 626005, TN, India

**Keywords:** breast cancer, image enhancement, biomedical engineering, internet of healthcare things

## Abstract

Breast cancer is the type of cancer with the highest incidence and global mortality of female cancers. Thus, the adaptation of modern technologies that assist in medical diagnosis in order to accelerate, automate and reduce the subjectivity of this process are of paramount importance for an efficient treatment. Therefore, this work aims to propose a robust platform to compare and evaluate the proposed strategies for improving breast ultrasound images and compare them with state-of-the-art techniques by classifying them as benign, malignant and normal. Investigations were performed on a dataset containing a total of 780 images of tumor-affected persons, divided into benign, malignant and normal. A data augmentation technique was used to scale up the corpus of images available in the chosen dataset. For this, novel image enhancement techniques were used and the Multilayer Perceptrons, k-Nearest Neighbor and Support Vector Machines algorithms were used for classification. From the promising outcomes of the conducted experiments, it was observed that the bilateral algorithm together with the SVM classifier achieved the best result for the classification of breast cancer, with an overall accuracy of 96.69% and an accuracy for the detection of malignant nodules of 95.11%. Therefore, it was found that the application of image enhancement methods can help in the detection of breast cancer at a much earlier stage with better accuracy in detection.

## 1. Introduction

It is reported that almost 24.2% of women with cancer in the world are affected by breast cancer each year, and 15% of deaths of female cancer-affected persons are from patients with this type of cancer [1]. According to the World Health Organization (WHO), 2.3 million women were diagnosed with breast cancer in 2020, and a mortality of 685,000 was reported [2]. In addition, as per the study in [3], it is anticipated that breast cancer cases will be increasing over the years and reach around 27 million in 2030.

Some patients have breast cancer cells but are asymptomatic, so frequent screening plays a vital role in detecting breast cancer before it becomes worse and progresses to subsequent stages [4]. To detect breast cancer, imaging tests can be used as well as biopsy, however, the biopsy is considered an invasive method, while imaging tests are more conservative [5].

Among the imaging tests, the most commonly used breast cancer detection techniques are mammogram, ultrasound and MRI [6,7]. Although imaging tests are fundamental in the identification and diagnosis of breast cancer, on several occasions, the tests are affected by noise, low contrast and other factors that impair the images and make it difficult to diagnose efficiently [8,9].

Due to the challenges encountered in the early stage of accurate diagnosis of breast cancer, industry and academia have been involved in active research with the aim of proposing computational tools capable of performing a diagnosis automatically [10].

The concept of computer-aided diagnosis (CAD) is being increasingly used to assist in the medical analysis of various diseases, such as, for example, vertebrae segmentation [11], diagnosis of Parkinson’s disease [10,12], perception of the dynamics of blood flow to from static CT angiography images [13], detection and classification of multiclass skin lesions via teledermatology [14], EEG-based BCI rehabilitation [15], recognition and detection of atrial fibrillation [16], detection of pulmonary nodules on CT scans [17], classification of oral cancer [18], as well as addressing security and efficient authentication for IoT applications in the medical field. In addition, research has been conducted on ways to improve the images [19,20,21], segment the parts of interest [22,23,24], and classify the nodes [25,26,27].

Thus, this research has the general objective of developing a robust platform to compare and evaluate image improvement methods in breast ultrasounds, classifying them into benign, malignant, and normal images. For this, we sought to apply novel methods of image enhancement; compare the methods applied; evaluate their performance through quality metrics; classify the images into three groups: normal, benign and malignant; and, finally, evaluate and validate the models used.

The main contributions of this research can be observed below:

- Development of a comparative analysis on the evaluation of breast cancer image enhancement methods to improve the accuracy in the detection of malignant tumors;
- Comparing different image enhancement techniques and classification techniques focused on breast tumor;
- Validating the results through statistical evaluations and estimating a better strategy for pre-screnning of tumors;
- Providing an online processing tool for breast cancer detection for early diagnosis and treatment.

Therefore, this work is structured in four sections. The first section deals with the introduction, addressing the contextualization, issues, objectives, and contributions. Then, the second section presents the methods incorporated for the development of the research. The third section addresses the results obtained in relation to image improvement and classification. Finally, the last section summarizes the work with conclusions and notes on future work.

## 2. Materials and Methods

**Figure 1:**
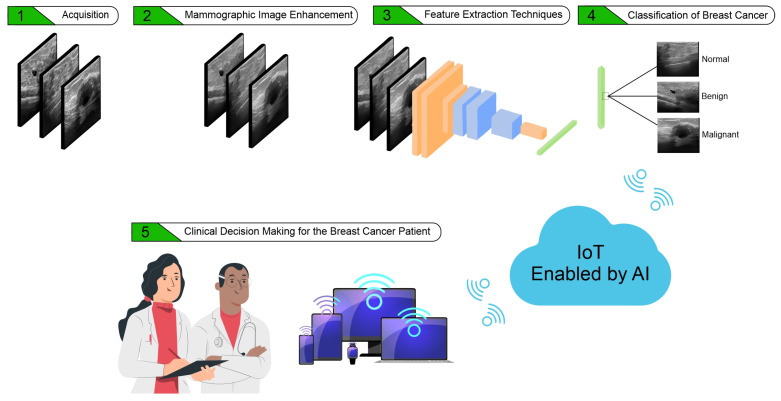
Sequence of stages involved in the proposed clinical decision-making system.

In this section, the application steps of the proposed system will be presented, covering the web platform, the image enhancement algorithms, the feature extraction method, the classifiers, as well as the validation metrics, and the experimental settings used in this study. Figure 1 shows the sequence of stages involved in the automated pre-screening of breast tumors using the proposed clinical decision-making system.

From the perspective of providing an interactive environment for screening tumor-affected patients, the proposed Web platform was built using HTML 5 and CSS 3 for the front-end interactive part with users, and the Flask micro-framework as a fast and lightweight development tool for the back-end design [28,29]. It is responsible for performing all the image enhancement processes, feature extraction [30], classification and metrics calculation, which will be discussed in the sections below.

Considering the functioning of the platform, the acquisition will be performed from the user uploads that include the breast ultrasound images with either png, jpg, jpeg or gif extensions and channelize them for cloud processing. The enhanced image features, the generated images, as well as their associated metrics, will be archived in a folder for further analysis and possible improvement in the training phases of the presented classification algorithms [31].

Subsequently followed by the enhancement, the generated images will be processed by the feature extractors and classified as normal, benign or malignant, and the metrics related to these processes will also be recorded in variables in the back end. Once all the results are calculated, the image storage path and the metric values are stored in a JSON file, which will be tracked by the front-end Flask framework, where the user can choose their visualization of interest.

### 2.1. Database Description

**Figure 2:**
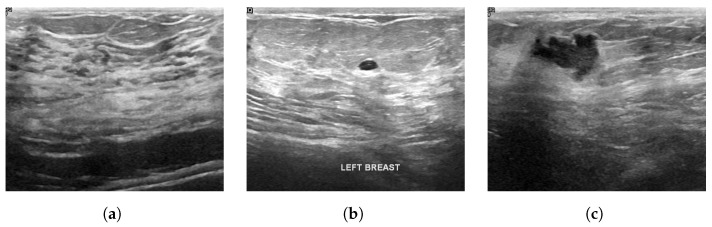
Examples of images of each class. (**a**) Normal (**b**) Benign (**c**) Malignant.

The chosen dataset of breast ultrasound images used in this study was proposed in [32], which includes data from 600 female patients aged between 25 and 75 years. The dataset comprises a total of 780 gray scale images separated into three classes: normal, containing a total of 133 images; benign, containing 437 images; and, malignant, containing 210 images. The images present in the dataset have an average dimension of 500 × 500 pixels, in PNG format. Figure 2 shows examples of ultrasound images for each class, normal image, image with benign nodule, and image with nodule and malignant. Table 1 lists the statistics on the count of the number of images in each category considered for the analysis.

#### Data Augmentation

In order to increase the number of base images, the Data Augmentation technique was used. The transformations used for this analysis were shear, rotation, horizontal translation (x-axis) and vertical translation (y-axis), with their corresponding values in degrees considered for transformation as 15${}^{{}^{\circ}}$, 15${}^{{}^{\circ}}$, 10${}^{{}^{\circ}}$ and 10${}^{{}^{\circ}}$, respectively. For every individual image, the type of transformation used is randomly chosen and they were performed thrice. Thus, the base dataset is augmented with triple the number of images of various transformations, and the corpus of images in the dataset will contain both the original images and the augmented images with applied transformations.

### 2.2. Image Enhancement Techniques

The methods used for image enhancements are categorized into traditional methods and methods based on Deep Learning, as shown below.

#### 2.2.1. Bilateral

The two-sided method proposed by [33], provides a traditional, iterative, local and simple strategy that smoothes images in order to preserve edges, through a non-linear combination of values from nearby images. The bilateral method combines shades of gray or colors based on their geometric proximity and photometric similarity, preferring close values to distant values, both in range and subject, to be within the domain.

#### 2.2.2. Histogram Equalization

Histogram equalization (HE) is another traditional method of image enhancement. Generally, histogram equalization increases the overall contrast of the images, especially when the used image data are represented by close contrast values, thus, it is a non-linear extension of the image, where it redistributes the pixel values and, within of a certain range of grayscale, the number of pixels is almost the same [34].

#### 2.2.3. Total Variance

The total variance (TV) method proposed by [35] is treated as another traditional image enhancement method. Here, the authors propose a new algorithm for minimization and total variance applications. It is treated as a very fast method and can be used to solve the challenges in noise reduction and zooming, in addition to ensuring better proof of convergence.

#### 2.2.4. Low-Light Image Enhancement via Illumination Map Estimation

The Low-Light Image Enhancement via Illumination Map Estimation (LIME) method is a simple low-light image enhancement method proposed by [36]. In this method, the illumination of each pixel is initially estimated individually, in order to find the maximum value for each component of the image, the R, G and B. The initial illumination map was refined by imposing a previous structure on it, such as the final lighting map, and thus, with a well-built lighting map, enhancement can be achieved [36].

#### 2.2.5. Exposure Fusion

In the work by Ying et al., in [37], the authors developed a new image contrast enhancement algorithm using an exposure fusion framework. This method is used for low-light images, where a weight matrix is designed for image fusion using lighting estimation techniques. With the model proposed by the authors, the camera response is used to synthesize multiple exposure images and, subsequently, it is used to estimate the best exposure ratio, such that the synthetic image is more exposed in the regions where the input image was underexposed. Thus, the input image and synthetic image are merged according to the weight matrix to achieve the expected enhancement result [37].

#### 2.2.6. Gamma Correction

The method proposed by the authors in [38] addresses contrast enhancement of glow-distorted images by enhanced adaptive gamma correction. The authors developed an improved adaptive gamma correction technique that utilizes a new negative image strategy to perform image contrast of bright images, employing truncated cumulative distribution function modulated gamma correction to enhance the faint ones, thus, distortion in the structure and challenges in local enhancement can be alleviated effectively.

#### 2.2.7. Light-DehazeNet

In the work of [39], a new lightweight CNN architecture for image defogging is proposed. The Light-DehazeNet (LD-Net) jointly estimates both the transmission map and atmospheric light using a transformed atmospheric scattering model. In breast ultrasound images with high-density impulse noise, the LD-Net ensures to realize quick and effective means of image denoizing.

#### 2.2.8. Zero-Reference Deep Curve Estimation

The paper proposed by [40] presents a Zero-Reference Deep Curve Estimation (Zero-DCE) as a new method that formulates light enhancement strategy as one of the image-specific curve estimation tasks using a deep neural network. It is highly instrumental in pixel-wise and high-order curve estimation and assists in adjusting the dynamic range of the input image. Further, the non-reference loss functions used for implementing the Zero-DCE help to realize an intuitive and simple nonlinear curve mapping.

#### 2.2.9. Low-Light Image Enhancement with Normalizing Flow

The research proposed by [41] addresses a new flow-based low-light image enhancement with normalizing flow method to accurately learn global image properties as well as local pixel correlations by modeling distributions over images normally exposed. It establishes the mapping relationship of low-light images from one to many by considering the conditional distribution. This LLFLOW technique helps to achieve enhanced images with better illumination, rich colors, and less noise, as well as artifacts.

### 2.3. Data Extraction

CNNs have been widely used for various purposes and applications [42,43,44,45]. To extract features from the images, we used the Resnet CNN proposed by the authors in [46], which possesses a residual learning structure that facilitates the training of substantially deeper networks. This type of network has greater ease of optimization and can gain accuracy even with considerably greater depth. Several methods have been developed based on Resnet, such as [47,48,49,50], which were observed to eliminate noise inference in the images, and assist in identifying even complex features with better accuracy.

In terms of the Resnet architecture, the vast majority of convolutional layers have 3 × 3 filters and follow two basic rules. Firstly, the layers have the same number of filters for the same output feature map size. Secondly, the number of filters is doubled if the feature map size is reduced by half, to preserve per-layer time complexity. Subsequently, the downsampling is performed directly by convolutional layers. It possesses a global averaging pool layer and a 1000-way fully connected layer with softmax, with a total of 34 weighted layers [46].

### 2.4. Classification Methods

For this study, the fully connected layer of the network was eliminated and only the core Resent50 was used for performing the feature extraction. Subsequently, to classify the data extracted by Resnet50, the MLP, SVM and KNN classifiers were used.

#### 2.4.1. Multi-Layer Perceptron

MLP is an unsupervised learning technique that consists of an artificial neural network based on forward-feeding biological neurons that has three types of layers, the input layer to collect the input data, the output layer that gives the decision over the input data, and the hidden layer, which lies between the input layer and the output layer [51,52].

At least one hidden layer is added to the MLP, and there can be numerous hidden layers. With the exception of the input layer, each node is a neuron with a nonlinear activation function [53].

#### 2.4.2. Support Vector Machine

The support vector machine (SVM) is based on the statistical learning theory [54]. SVM is a supervised machine learning technique that builds a set of hyperplanes in a high-dimensional space, and good separation of the hyperplanes is achieved when it is obtained based on the greatest distance to the closest training data point of any class [55].

#### 2.4.3. k-Nearest Neighbor

The k-Nearest Neighbor algorithm is a supervised machine learning regression and classification technique [56]. The idea of kNN is that the point *k* closest to the sample to be tested is usually found through the Euclidean distance [57]. If most of the points *k* belong to the same category as the sampled, this sample will be classified in the same class [58].

### 2.5. Statistical Metrics

The metrics used for analysis were both to evaluate the quality of the images, as well as to evaluate the classification accuracy.

#### 2.5.1. Quality Metrics

The quality of assessment process was ensured through several aspects that can be observed through the following metrics:

- RMSE: The Root Mean Square Error (RMSE) is a metric that considers the number of errors between two sets of data. In this metric, the closer to zero, the more accurate the observed forecast results [59]. Thus, a comparison will be made with the two breast ultrasound images, the original and the image after applying the improvement algorithms.
  (1) $$RMSE=\sqrt{\frac{\sum_{i=1}^{n}{\left(P{}_{i}-O{}_{i}\right)}^{2}}{n}}$$
  where $P{}_{i}$ is the actual value of the data, $O{}_{i}$ is the predicted value, *n* is the number of data and $\sum_{}^{}$ is the total number of values.
- CNR: The contrast-to-noise ratio (CNR) is a metric to measure the contrast of images. It enables us to analyze the difference in contrast between the nodules and the other regions in the breast ultrasound images [60].
  (2) $$\mathrm{CNR}=\frac{|\mu_{i}-\mu_{o}|}{\sqrt{\sigma_{i}^{2}+\sigma_{o}^{2}}},$$
  where,
  (3) $$\begin{array}{cc} \sigma_{i}\; & =E\{(∥s_{i}{∥}^{2}-\mu_{i}{)}^{2}\}, \end{array}$$
  (4) $$\begin{array}{cc} \sigma_{o}\; & =E\{(∥s_{o}{∥}^{2}-\mu_{0}{)}^{2}\}, \end{array}$$
  which are the variation of signal strength inside and outside the target area, respectively.
- AMBE: The Absolute Mean Brightness Error (AMBE) is a metric that evaluates the difference between the average intensity level of the enhanced ultrasound image and the average intensity level of the original image [61].
  (5) $$AMBE=\left|I(y)-I(x)\right|$$
  where I(y) is the average intensity level of the enhanced image and I(x) is the average intensity level of the original image.
- AG: The Average Gradient (AG) is a metric that represents the clarity of the breast ultrasound image, reflecting the image’s ability to express contrast details between the nodule and the other regions [62].
  (6) $$AG=\frac{1}{MxN}\sum_{i=1}^{M}\sum_{j=1}^{N}\sqrt{\frac{{\left(\frac{\partial f}{\partial x}\right)}^{2}+{\left(\frac{\partial f}{\partial y}\right)}^{2}}{2}},$$
  where *M* and *N* are the width and height of the image, $\left(\frac{\partial f}{\partial x}\right)$ and $\left(\frac{\partial f}{\partial y}\right)$ refers to the horizontal and vertical gradients.
- PSNR: Peak Signal-to-Noise Ratio (PSNR) is a metric that evaluates the relationship between the maximum value of the measured signal and the amount of noise that affects the signal of breast ultrasound images [59].
  (7) $$PSNR=20log_{10}\left(\frac{MAX_{f}}{\sqrt{MSE}}\right)$$
  where $MAX_{f}$ is the maximum value and $\sqrt{MSE}$ is the result of the RMSE.
- SSIM: The Structural Similarity Index (SSIM) is one of the quality assessment metric used to measure the visual changes and similarity between two images, by performing quality assessment and comparing the structural characteristics, which is described through the structural similarities [59]. In this way, it helps to analyze the similarity between the original breast ultrasound image and the image after applying the image improvement algorithm.
  (8) $$SSIM\left(x,y\right)=\frac{\left(2\mu_{x}\mu_{y}+c_{1}\right)\left(2\sigma_{xy}+c_{2}\right)}{\left(\mu_{x}^{2}+\mu_{y}^{2}+c_{1}\right)\left(\sigma_{x}^{2}+\sigma_{y}^{2}+c_{2}\right)}$$
  where, with an image I(x,y) and $\mu_{x}$ being the average value for *x* or luminance *x*, $\mu_{y}$ being the average value for *y* or luminance *y*, $\sigma_{y}$ the contrast value for *y*, $\sigma_{x}$ the contrast value for *x*, $c_{1}$ and $c_{2}$ being two variables used to stabilize the division if the divisor is 0.

#### 2.5.2. Rank Metrics

The classification of clinical data presents a few challenges, among them, two of which are primary ones to be addressed. The first is the unbalanced dataset, where a greater number of cases have negative diagnoses than positive diagnoses. In the second one, the main interest is to accurately classify the positive case of the disease, since false positives do not cause great damage while false negatives can result in a delay in treatment, consequently increasing the difficulty of an early diagnosis.

To measure the accuracy of the method, the confusion matrix is used as a basis, using the evaluation metrics of True Positive (VP), False Positive (FP), True Negative (VN) and False Negative (FN).

**Figure 3:**
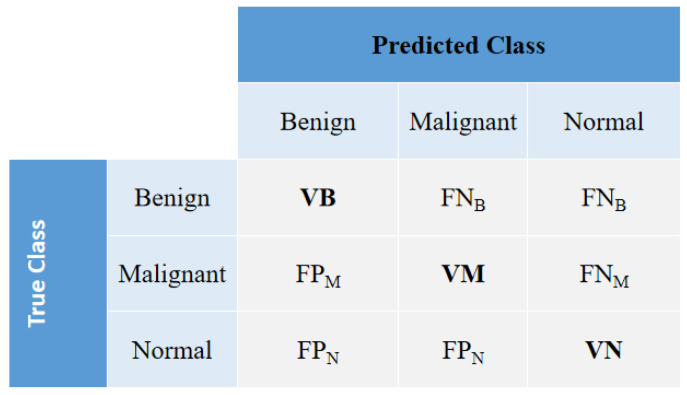
The confusion matrix highlighting the performance accuracy of the classification of mammogram images, with the diagonal elements in bold representing the true prediction labels.

As this search has three classes, benign, malignant and normal, the confusion matrix can be described as shown in Figure 3.

In this way, we have:

- True Positive class Benign (VB): VB occurs when in the actual dataset, class Benign was correctly predicted as class Benign.
- True Positive Malignant class (VM): The VM occurs when in the actual dataset, the Malignant class was correctly predicted as the Malignant class.
- True Positive Normal class (VN): The VN occurs when in the actual dataset, the Normal class was correctly predicted as the Normal class.
- False Negative (FN): FN occurs when in the actual data set, the class we are trying to predict was predicted incorrectly. That is, when it was supposed to be cancer and was diagnosed as non-cancer.
- False Positive (FP): FP occurs when in the actual dataset, the class we are trying to predict was predicted incorrectly. That is, when it was supposed to be non-cancer and was diagnosed as cancer.

For this, five metrics were used to evaluate the results considering these questions, namely: Accuracy (AccGlobal), F1-score (F1score), benign class hit rate (Benign), malignant class hit rate (Malignant), and normal class hit rate (Normal).

- Accuracy: This is the general probability of success, which shows the global success rate considering the analyzed classes. Thus, it takes into account the hits of the three classes under all hits and misses.
  (9) $$AccGlobal=\frac{VB+VM+VN}{VB+VM+VN+FN+FP}$$
- F1-score: It is the harmonic average between precision and recall. It is a commonly used metric to assess unbalanced data.
  (10) $$F1score=2\times \frac{Precision\times Recall}{Precision+Recall}$$
- Hit rate of benign class (Benign): This is the probability that a patient who has a positive diagnosis for benign actually has a benign nodule.
  (11) $$Benign=\frac{VB}{VB+FN_{B}+FN_{B}}$$
- Hit rate of Malignant class: This is the probability that a patient who has a positive diagnosis for malignant actually has a malignant nodule.
  (12) $$Malignant=\frac{VM}{VB+FP_{M}+FN_{M}}$$
- Hit rate of the Normal class (Normal): This is the probability that a patient who has a negative diagnosis for nodules actually does not have nodules.
  (13) $$Normal=\frac{VN}{VN+FP_{N}+FP_{N}}$$

### 2.6. Experimental Configuration

Initially, a Resnet50 (a CNN with 50 layers deep neural network) was implemented as a feature extractor, learning intrinsic patterns from images to identify breast cancer. For classification, the algorithms MLP, SVM and kNN were used. The dataset extracted by Resnet50 was divided into training data using k-fold Cross Validation with 20 fields. Hyperparameters were optimized through grid search. Relative to kNN, the number of neighbors ∈ [3, 10] and leaf size ∈ [10, 50]. The SVM parameters, the γ∈$\left[2^{-15},2^{-1}\right]$ and *C*∈$\left[2^{-5},2^{5}\right]$. As for MLP, the parameters were the number of hidden layers ∈ [1:5], the number of neurons per hidden layer ∈ [50:500], α∈ [000001.1], and learning rate ∈ [000001.0.9999].

The experiments were performed on a computing terminal with Windows 11 Operating System (a new version from Microsoft Corporation), i7 11800H processor, 8-core, 24Mb Cache, 4.6GHz, 11th generation, 16Gb of DDR4 RAM, 3200 MHz, and NVIDIA Geforce RTX 3060 video card (a Graphical Processing Unit from NVIDIA Corporation), 6GB GDDR6.

## 3. Results and Discussion

### 3.1. Image Enhancement

With the application of the enhancement algorithms on the ultrasound images, the processing duration of each approach was summarized as shown in Table 2. It is evident from the statistics that the Bilateral and LIME algorithms consume much longer processing time compared to the other algorithms, particularly the Bilateral with 1344 min. The Gamma correction method was the one that consumes the shortest processing time of 2.8 min.

From the perspective of the processing times of the algorithms, the metrics presented in Table 3 is used to assess the quality of the obtained images. It can be seen that in relation to the RMSE metric and the AMBE metric, the algorithm that obtained the best results was the TV with 0.0227 and 0.0017, respectively. The bilateral algorithm achieved a result close to the TV, with RMSE of 0.0329 and AMBE of 0.0063. The HEP and LIME algorithms achieved inferior results both in the RMSE metric and in the AMBE metric among the other algorithms.

Regarding the CNR metric, the algorithms that had the highest contrast–noise ratio were the HE and LIME methods, while the lowest contrast–noise ratio were the TV and Bilateral methods.

Analyzing the AG metric, which evaluates the change in the intensity of pixel values, it can be seen that the smallest change was for the Bilateral and LIME methods, both with 0.0, while the Ying method was the one that caused the greatest change in intensity. When analyzed from the perspective of the PSNR metric, the TV method achieved the best result with 81.0792, followed by the Bilateral method with 77.8054. The Gamma algorithm achieved the worst result compared to the others, with 36.7292.

With the conception of the SSIM metric, the Ying algorithm achieves the closest result to the original image with 0.9394, while LDNet being the most distant method with 0.3705, followed by LIME with 0.3778. The Gamma, Bilateral, TV and LLFLOW methods achieved the SSIM value above 0.80.

Considering all the metrics, the TV method achieved a good performance in three of the seven analyzed metrics. The Bilateral method achieved the best result in the AG metric and also achieved good results in the RMSE, AMBE and PSNR metrics.

**Figure 4:**
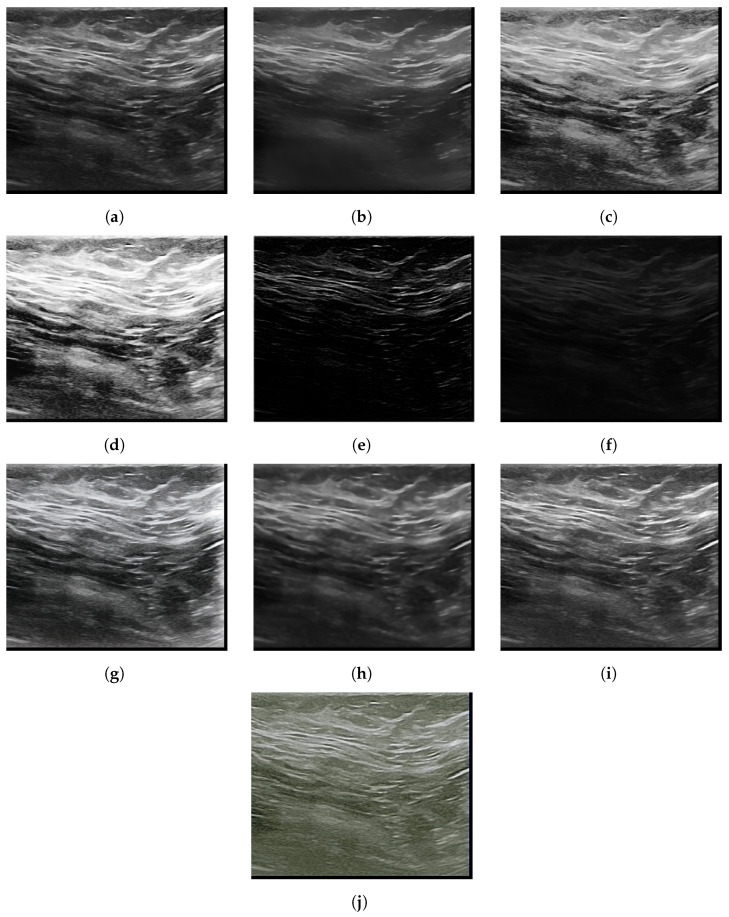
Normal highlighted mammogram images (**a**) Original (**b**) Bilateral (**c**) Gamma correction (**d**) HE (**e**) LDNET (**f**) LIME (**g**) LLFLOW (**h**) TV (**i**) Ying (**j**) Zero-DCE.

**Figure 5:**
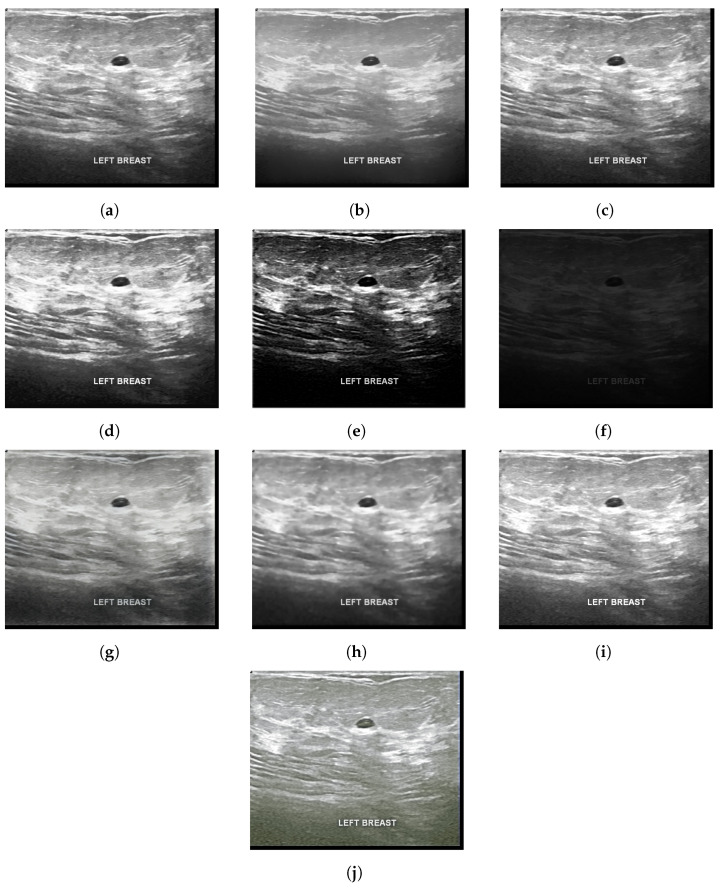
Benign highlighted mammogram images (**a**) Original (**b**) Bilateral (**c**) Gamma correction (**d**) HE (**e**) LDNET (**f**) LIME (**g**) LLFLOW (**h**) TV (**i**) Ying (**j**) Zero-DCE.

**Figure 6:**
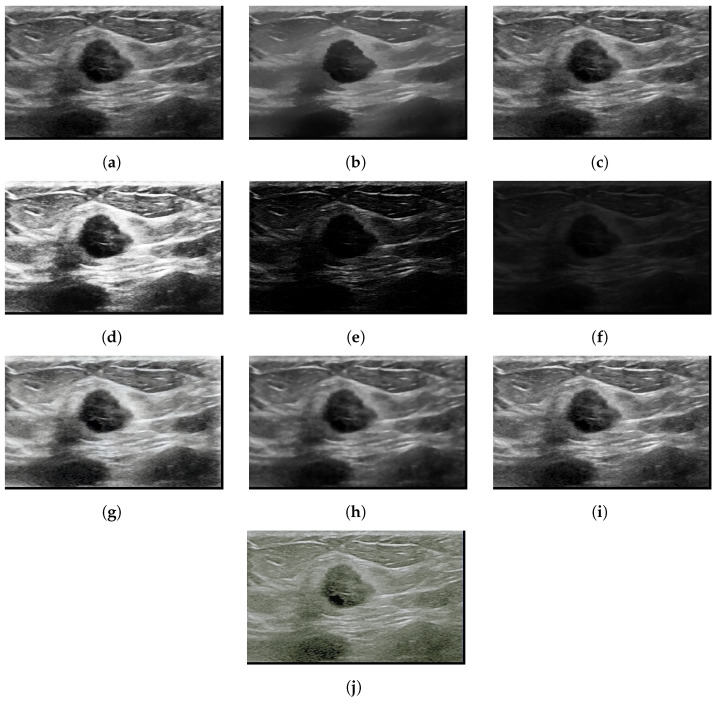
Malignant highlighted mammogram images (**a**) Original (**b**) Bilateral (**c**) Gamma correction (**d**) HE (**e**) LDNET (**f**) LIME (**g**) LLFLOW (**h**) TV (**i**) Ying (**j**) Zero-DCE.

With the application of image enhancement algorithms, it can be seen in Figure 4, Figure 5 and Figure 6 the elimination of some noise that presents the same characteristics of the region of interest. It is noted that the TV and Bilateral algorithms, which presented better results in quality metrics, have images with less noise compared to the other algorithms, which can facilitate better classification accuracy.

### 3.2. Classification

From the data extracted with Resnet50, the results were obtained using the MLP, kNN and SVM algorithms for the metrics of global accuracy (ACC Global), benign success rate (Benign), malignant success rate (Malignant), hit rate of normals (Normal) and F1-score. Furthermore, presented in Table 4 are the training and testing times for each algorithms for image enhancement, considering the MLP, kNN and SVM classifiers.

It is worth noting that the kNN classifier provides the shortest training time in relation to the other algorithms. Further, the combination of the HE enhancement approach with the kNN achieved the shortest training time. The MLP classifier, despite being the one that takes more training time, in relation achieves the shortest test times. Moreover, it is observed that the TV method in association with the MLP achieved the shortest test time.

In Table 5 are presented the metrics for each image enhancement algorithm, as well as for the original base. Analyzing the enhancement algorithms for each classifier individually, we observe that for the MLP classifier, the algorithm that obtained the best accuracy result was the TV algorithm with 95.54%, being considered statistically similar to the Bilateral method that reached an accuracy rate of 95.54%.

For the kNN classifier, the algorithm that obtained the best results in terms of both accuracies and hit rate of images with malignant nodules (malignant) was LDNET with 83.23% and 76.90%, respectively. For the SVM classifier, the algorithm that obtained the best global accuracy was the Bilateral one with 96.69%. The TV algorithm delivers an accuracy rate of 96.66%, by considering the Wilcoxon method, which is statistically equal to the Bilateral method.

Analyzing in general, considering the best combination between the image improvement algorithm and classifier, the one that delivers the better results with balanced trade-offs was the Bilateral algorithm in association with the SVM classifier.

Although global accuracy is a valid metric, the accuracy rate in the classification of images with malignant nodules (malignant) is fundamental for this analysis, as it is more clinically relevant. Thus, it can be seen that the Bilateral algorithm with the SVM classifier for the malignant metric achieved the best result among all combinations, with 95.11%.

Taking into account the training and testing times along with their the hit metrics, it is clear that despite the kNN training time being the shortest, it was the algorithm that delivers the inferior results for all image enhancement methods. The SVM that achieved neither the shortest, nor the longest training and test times assists in providing better classification accuracy.

**Figure 7:**
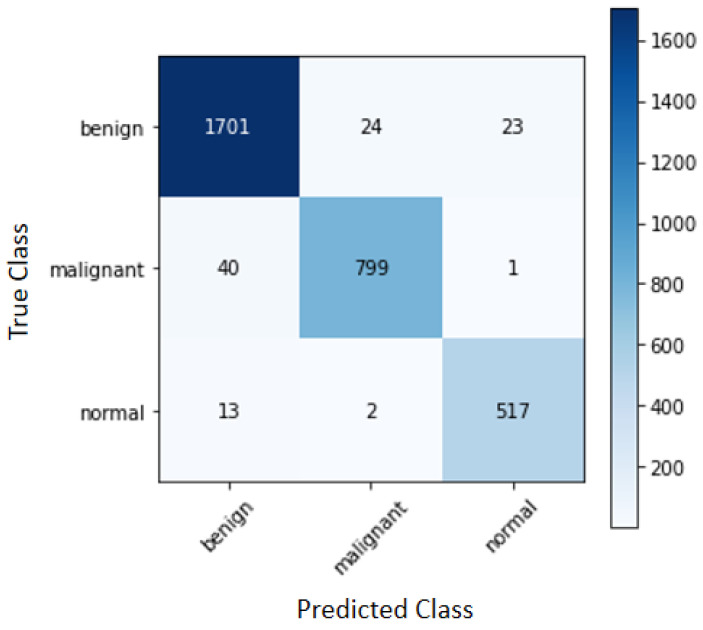
Confusion matrix for the combination of the Bilateral algorithm with the SVM classifier.

Considering only the best combination that was targeted for achieving the best result of global and malignant accuracy, Figure 7 shows the confusion matrix for the combination of the bilateral method with the SVM classifier.

It is evident from the analysis that the benign class had 1701 correctly classified, while the malignant and normal had 799 and 517, respectively. The benign class possesses more errors than the malignant class, as these two classes contain nodules in their images. A similar situation occurred with the malignant class as well, as there were more errors in the benign class. In the normal class, there are more errors than in the benign class, which could also be observed in comparison with the normal class, which comes very close to the error of the malignant class that has the nodule.

One of the possible explanations is that, although normal images do not have nodules, there are still regions in the ultrasound image that may have similarities with nodules. The characteristics of benign nodules are that they are well-defined and regular, whereas malignant nodules are larger and asymmetrical in shape. Thus, it may be observed that some of the regions in the normal images resemble the nodules in the benign images.

**Figure 8:**
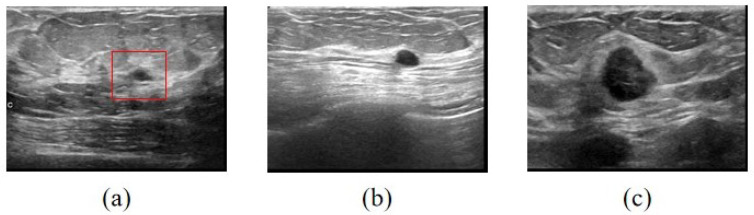
Database images. (**a**) Normal image with region marked in red that resembles a benign nodule. (**b**) Image with a benign nodule. (**c**) Image with malignant nodule.

In Figure 8, it is possible to verify in (a) that the region marked in red has a similarity with the benign nodule, even though it has a lighter texture. In Figure 8b,c, one can see the shapes of both benign and malignant nodules through their visual differences.

Analyzing the complete confusion matrix, it is noted that there were only 48 false negatives and 15 false positives. These false negatives and false positives are the ones that can cause the most inconvenience and delays in medical treatments and diagnoses, so minimizing these false positives is of great importance. Thus, observing Table 3, which presents the image quality metrics, and Table 5, which presents the classification metrics, it is clear that the algorithms that obtained better results in the metrics of quality were the ones that achieved the best results in the ranking metrics. The TV method and the Bilateral method stood out in terms of the quality metrics and achieved reasonable rates in the classification.

More stability and consistency in the results of the proposed image enhancement techniques were proved with better precision in the earlier stage detection of breast cancers. Particularly, the novel approaches in the pre-processing stages of the breast ultrasound image using different image enhancement algorithms guarantees stabilized outcomes on the chosen dataset. Therefore, it can be verified that good pre-processing, making improvements in the images, can considerably benefit the classification results.

### 3.3. Online System/Web Interfaces

**Figure 9:**
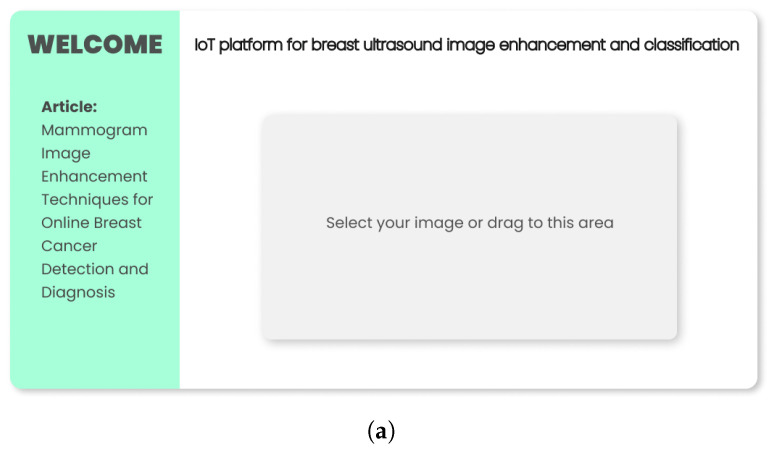

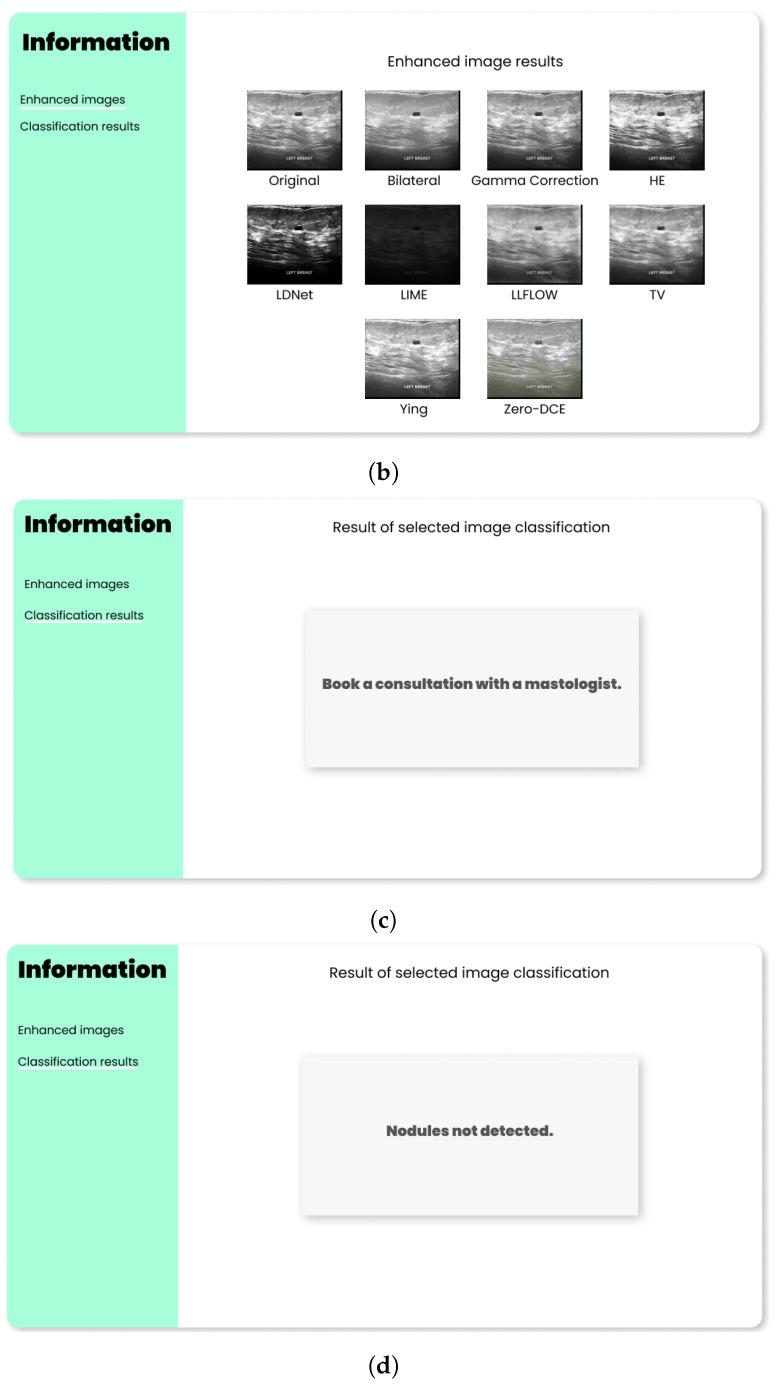
Online web platform interfaces modules. (**a**) Main interface module (**b**) Module with images enhancement (**c**) Classification module with nodules (**d**) Nodule-free sorting module.

The online platform for automatic pre-screening of breast tumors basically consists of four modules: the main interface, the interface with enhanced images, the classification interface when there are nodules and the classification interface when there are no nodules, as specified in Figure 9.

Figure 9a shows the main interface module, where it will be possible to upload the breast tumor images to be analyzed. After uploading, the web portal redirects users to the second interface. This module presents a menu-driven interface with the users, which will be capable of analyzing the pre-processed images by the image enhancement methods, as well as the classification result. When clicking on the “Enhanced images” button in the menu, the users will be redirected to the interface containing the specified image with the application of image enhancement methods, as shown in Figure 9b.

By clicking on “Classification results” in the menu, the users will be presented with a message. If there is a nodule, a message will be displayed to consult a mastologist (as shown in Figure 9c), if there is no nodule, the user will be informed that no nodule was found in the image (as shown in Figure 9d).

## 4. Conclusions and Future Work

This research proposed the development of an online platform to compare and evaluate methods of enhancing breast ultrasound images, classifying them as benign, malignant and normal. For this purpose, a database containing 437 benign images, 210 malignant images and 133 normal images were used. Due to the small number of images, various data augmentation techniques were used. To extract the characteristics of these images, Resnet50 was used. For these extracted data, the SVM, kNN and MLP algorithms were used to perform the classification.

It was found through the experimental results that the application of appropriate image enhancement methods can improve classification performance. In addition, the algorithms that obtained better results in the metrics for achieving better image quality provided the best results in the classification.

It is observed that the Bilateral enhancement method together with the SVM classification algorithm obtained the highest hit rate, both for global accuracy and for accuracy in the detection of malignant tumors. Therefore, it is noted that the application of image enhancement methods has significant relevance to the diagnosis of breast cancer.

### Future Work

In this context, the following future works are intended to open up better opportunities for the researchers:

- Developing novel light image enhancement strategies specific for breast cancer, considering the applied generic enhancement algorithms;
- Embedding such new enhancement approaches in specific hardware modules with the possibility of interacting with the cloud;
- Building 3D reconstruction models to perform the volumetric quantification of the nodules;
- Building a web dashboard to analyze the experiments as well as the 3D reconstruction with better visualizations.

## Figures and Tables

**Table 1:** Base data of the images considered for analysis.

Information	Amount	Percent
Normal Images	133	17.05
Benign Images	437	56.03
Malignant Images	210	26.92

**Table 2:** Comparison of processing time taken by image enhancement algorithms (in minutes).

Algorithms	Processing Time (minutes)
Bilateral	1344.5736
Gamma correction	2.8820
HE	7.1598
LDNet	6.7305
LIME	651.8196
LLFLOW	9.2683
TV	17.3219
Ying	8.8536
ZDCE	9.5254

**Table 3:** Quality metrics for image enhancement algorithms.

Algorithms	RMSE	CNR	AMBE	AG	PSNR	SSIM
Bilateral	0.0329	0.0317	0.0063	0.0	77.8054	0.8726
Gamma correction	0.0936	0.4060	0.0763	0.0363	36.7292	0.8924
HE	0.2391	1.1135	0.2161	0.0364	61.2157	0.6347
LDNet	0.2360	0.9508	0.1941	0.0361	60.7778	0.3705
LIME	0.2801	1.0946	0.2272	0.0	59.4688	0.3778
LLFLOW	0.1342	0.5578	0.1069	0.0184	66.3159	0.8116
TV	0.0227	0.0088	0.0017	3.86e-05	81.0792	0.8569
Ying	0.0691	0.3125	0.0621	0.1676	71.6003	0.9394
ZDCE	0.1334	0.6059	0.1189	0.0362	65.6991	0.7858

**Table 4:** Training and testing times of image enhancement algorithms and the classifiers.

Algorithms	Times (seconds)	MLP	kNN	SVM
Original	Training	161.605	0.105	109.754
	Test	0.036	1.119	5.582
Bilateral	Training	0.1076	0.107	114,538
	Test	0.034	1.105	5.741
Gamma correction	Training	142.177	0.106	113.221
	Test	0.039	1.231	5.801
HE	Training	164.593	0.104	117.841
	Test	0.033	1.176	5.906
LDNET	Training	197.522	0.106	119.007
	Test	0.040	1.143	6.101
LIME	Training	158.675	0.105	110.196
	Test	0.032	1.193	5.622
LLFLOW	Training	120.524	0.106	114.953
	Test	0.032	1.205	5.803
TV	Training	141.409	0.106	115.173
	Test	0.031	1.214	5.900
Ying	Training	156.628	0.105	115.086
	Test	0.033	1.194	5.874
Z-DCE	Training	169.422	0.105	123.348
	Test	0.037	1.122	6.170

**Table 5:** Metrics for image enhancement algorithms.

Algorithms	Metrics	MLP	kNN	SVM
Original	ACC Global	94.99 ± 1.65	77.85 ± 3.30	96.50 ± 1.82
	Benign	97.08 ± 1.50	76.44 ± 4.98	97.42 ± 1.44
	Malignant	92.50 ± 3.21	71.90 ± 5.90	94.40 ± 2.94
	Normal	92.15 ± 7.50	91.91 ± 5.87	96.80 ± 3.99
	F1-score	94.96 ± 1.69	78.57 ± 3.20	96.49 ± 1.56
Bilateral	ACC Global	95.54 ± 1.36	79.07 ± 2.45	**96.69 ± 1.56**
	Benign	96.68 ± 1.91	78.78 ± 3.06	97.31 ± 1.94
	Malignant	92.61 ± 3.44	73.21 ± 5.92	95.11 ± 2.86
	Normal	96.41 ± 4.41	89.31 ± 5.65	97.18 ± 3.54
	F1-score	95.53 ± 1.37	79.70 ± 2.41	96.69 ± 1.56
Gamma correction	ACC Global	94.93 ± 1.59	77.91 ± 2.66	96.47 ± 1.33
	Benign	96.74 ± 1.57	77.17 ± 4.08	97.54 ± 1.31
	Malignant	91.90 ± 3.86	70.83 ± 5.52	93.92 ± 3.32
	Normal	93.83 ± 5.90	91.54 ± 6.45	97.00 ± 3.66
	F1-score	94.91 ± 1.61	78.72 ± 2.65	96.46 ± 1.35
HE	ACC Global	95.28 ± 1.71	78.07 ± 2.62	96.31 ± 1.38
	Benign	96.45 ± 2.01	78.20 ± 4.21	97.25 ± 1.50
	Malignant	92.97 ± 3.87	75.23 ± 5.80	94.40 ± 2.84
	Normal	95.15 ± 4.55	82.15 ± 7.88	96.26 ± 3.51
	F1-score	95.27 ± 1.71	78.45 ± 2.54	96.30 ± 1.39
LDNET	ACC Global	94.96 ± 1.96	83.23 ± 2.54	96.31 ± 1.52
	Benign	96.90 ± 1.81	84.61 ± 3.07	97.19 ± 1.59
	Malignant	93.09 ± 4.63	76.90 ± 5.48	94.40 ± 3.55
	Normal	91.50 ± 7.48	88.73 ± 6.95	96.45 ± 4.60
	F1-score	94.93 ± 1.99	83.44 ± 2.46	96.30 ± 1.53
LIME	ACC Global	95.51 ± 1.46	81.57 ± 2.57	96.47 ± 1.24
	Benign	96.91 ± 1.72	82.21 ± 3.45	97.25 ± 1.36
	Malignant	92.38 ± 3.49	75.00 ± 5.90	94.16 ± 3.14
	Normal	95.88 ± 3.71	89.87 ± 6.47	97.56 ± 2.45
	F1-score	95.50 ± 1.46	82.00 ± 2.55	96.46 ± 1.24
LLFLOW	ACC Global	95.06 ± 1.66	75.96 ± 3.40	96.34 ± 1.07
	Benign	96.23 ± 2.25	75.74 ± 3.73	97.25 ± 1.32
	Malignant	92.49 ± 3.47	68.09 ± 6.79	93.69 ± 2.94
	Normal	95.34 ± 6.82	89.10 ± 6.33	97.57 ± 3.77
	F1-score	95.04 ± 1.66	76.75 ± 3.25	96.33 ± 1.07
TV	ACC Global	95.64 ± 1.41	77.33 ± 3.21	96.66 ± 1.66
	Benign	96.97 ± 1.57	73.97 ± 5.25	97.42 ± 1.57
	Malignant	93.09 ± 3.18	76.30 ± 5.02	94.88 ± 2.84
	Normal	95.31 ± 4.38	90.05 ± 5.44	97.00 ± 3.86
	F1-score	95.63 ± 1.42	78.08 ± 3.06	96.66 ± 1.67
Ying	ACC Global	95.22 ± 1.56	78.14 ± 3.35	96.57 ± 1.39
	Benign	96.22 ± 1.76	76.90 ± 5.01	97.37 ± 1.44
	Malignant	93.80 ± 3.94	73.21 ± 7.25	94.64 ± 3.18
	Normal	94.18 ± 6.42	90.02 ± 5.97	96.99 ± 3.69
	F1-score	95.21 ± 1.58	78.93 ± 3.16	96.56 ± 1.39
Z-DCE	ACC Global	94.23 ± 2.18	76.89 ± 2.96	95.80 ± 1.35
	Benign	96.68 ± 1.56	78.56 ± 5.18	97.14 ± 1.46
	Malignant	92.14 ± 4.06	69.40 ± 5.54	93.09 ± 2.90
	Normal	89.52 ± 9.41	83.31 ± 7.36	95.70 ± 3.74
	F1-score	94.17 ± 2.25	77.25 ± 2.94	95.79 ± 1.35

## Data Availability

Not applicable.
